# Constraint Logic Programming approach to protein structure prediction

**DOI:** 10.1186/1471-2105-5-186

**Published:** 2004-11-30

**Authors:** Alessandro Dal Palù, Agostino Dovier, Federico Fogolari

**Affiliations:** 1 Dipartimento di Matematica e Informatica, Università di Udine. Via delle Scienze 206, 33100 Udine, Italy; 2 Dipartimento di Scienze e Tecnologie Biomediche, Università di Udine, P.le Kolbe 4, 33100 Udine, Italy

## Abstract

**Background:**

The protein structure prediction problem is one of the most challenging problems in biological sciences. Many approaches have been proposed using database information and/or simplified protein models. The protein structure prediction problem can be cast in the form of an optimization problem. Notwithstanding its importance, the problem has very seldom been tackled by Constraint Logic Programming, a declarative programming paradigm suitable for solving combinatorial optimization problems.

**Results:**

Constraint Logic Programming techniques have been applied to the protein structure prediction problem on the face-centered cube lattice model. Molecular dynamics techniques, endowed with the notion of constraint, have been also exploited. Even using a very simplified model, Constraint Logic Programming on the face-centered cube lattice model allowed us to obtain acceptable results for a few small proteins. As a test implementation their (known) secondary structure and the presence of disulfide bridges are used as constraints. Simplified structures obtained in this way have been converted to all atom models with plausible structure. Results have been compared with a similar approach using a well-established technique as molecular dynamics.

**Conclusions:**

The results obtained on small proteins show that Constraint Logic Programming techniques can be employed for studying protein simplified models, which can be converted into realistic all atom models. The advantage of Constraint Logic Programming over other, much more explored, methodologies, resides in the rapid software prototyping, in the easy way of encoding heuristics, and in exploiting all the advances made in this research area, e.g. in constraint propagation and its use for pruning the huge search space.

## Background

Notwithstanding the continuous improvement in predictive methods, witnessed every two years by the world wide CASP experiment [1,2], predicting the structure of a protein, given its sequence, is still in general beyond our capabilities. Brute force approaches, like exhaustive conformational searches or molecular dynamics simulations of the folding process, are precluded by the computing power available at present. Alternative, faster methods have been developed along two main lines:

1. assemblying the structure of a protein using structural fragments of similar sequences, available in the protein structure repository (the Protein Databank [3]), and later screening the feasibility of the resulting structures, using energetic criteria;

2. representing the protein chain by a highly simplified model which is, hopefully, treatable.

This second class of approaches is appealing in many respects [4]: first, the linkage between kinetics and thermodynamics of protein folding process and the basic intramolecular interactions is more easily addressable, because of the lesser number of variables. Second, the use of a simplified model agrees with the idea that details of atomic interactions between aminoacids are less important than the overall character of these interactions, because protein structure is flexible and can accommodate changes in the volume and shape of aminoacids much better than changes in their character (e.g. polar vs. hydrophobic [5]). Besides aiming at catching essential features of the protein folding process, simplified models have important computational advantages: generating and evaluating the energy of a conformation is efficiently done due to the reduced number of variables. A less evident benefit is that sampling (e.g. by molecular dynamics simulation or Monte Carlo methods) may be much more efficient due to the smoothness of energy surface due, once again, to the reduced number of degrees of freedom. Many lattice models have been used for simplified representation of proteins, up to date. Their capability of reproducing the secondary structure of proteins, as well as their relative arrangement has been reviewed by Godzik et al. [6]. A reasonable tradeoff between accuracy and the need to keep limited the number of base vectors is achieved by the face centered cubic (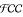) lattice studied by Toma and Toma [7]. In particular both *α*-helices and *β*-strands are modelled with a very low RMSD from standard regular structures. Lattice models have been used mainly for understanding general properties of proteins, rather than for real predictive tasks, although their use, especially in hierarchical protocols has been proposed and realized. In particular, the (210) lattice has been used successfully by Skolnick and Kolinski in prediction of a small beta protein [8] and many other useful applications have been reported since these earlier works (see e.g. for recent successful applications [9,10] and also the two recent reviews [4,11]). A deep analysis of realistic lattice models of proteins proposed so far is definitely out of the scope of the present work, but there are few aspects of lattice models of proteins which need to be mentioned. The successful application of a lattice model depends obviously on the efficiency in generating conformations and searching for local minima. This aspect is dealt in the present work using Constraint Logic Programming, and taking advantage of all theoretical and implementative developments that have been realized in this context. The approach (and related languages) has been very seldom applied in the context of protein modeling and it has not been used for realistic protein structural predictions, to the best of our knowledge. A different, but equally important, aspect concerns the reliability of the model itself and of the forcefield used to evaluate conformational free energy. This aspect will not be dealt with by this work. An appropriate forcefield must take into account both local propensities to adopt a particular secondary structure (which ultimately depend on aminoacids' covalent structure and bulkiness) and their tendency to be in contact (which ultimately depends on their physico-chemical character). Contact potentials have been derived by many groups (see e.g. [12,13]) based on the observed versus expected contacts stored in the database. A similar approach could be followed in order to derive a torsional potential in order to describe local conformational propensities. However, it is not obvious how these potentials should be derived for lattice models and how the two potentials are to be considered together. These problems are not investigated here. Rather we consider contact potentials previously derived by our group from statistical analysis of the database [13], which are expected not to be accurate for a lattice model, but nevertheless should be able to reproduce essential features of aminoacid interactions. The local propensity to adopt a particular secondary structure can be computed by predictive methods [14]. However, for the small peptides analyzed in this paper, the correct secondary structure is selected from the deposited structures for testing purposes.

*Constraint Logic Programming *(briefly, 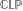) [15,16] is a declarative programming paradigm particularly well-suited for encoding combinatorial minimization problems. It is the natural merger of the two declarative paradigms known as *Constraint Solving *and *Logic Programming*.

One of the peculiar features of 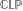 is the independence of the problem modeling and of the search's strategy. Problem modeling is based on traditional declarative programs in which one can use the built-in notion of *constraint*. Constraints are first-order formulas concerning variables that can assume values in some *domains*. The scheme is general. Various possible constraints and domains can be used. However, for combinatorial problems it is common to use *finite domain constraints*, namely arithmetic constraints between arithmetic expressions, where variables range over finite subsets of ℕ. Constraint Logic Programming over Finite Domains is known as 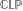
(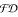). We briefly introduce this programming paradigm with a simple example. Let us consider three variables *X*, *Y*, *Z *that denote the number of possible items of some kind.

domain([*X*, *Y*, *Z*], 1, 10)

is a constraint that states that the three variables *X*, *Y*, *Z *have (finite) domain {1, 2, ..., 10}. Suppose we wish to state that the *weight *of each item of *X *is 3, of *Y *is 4, and of *Z *is 5 and the total weight of selected items must be less than or equal to 40. Moreover, we wish to state that the number of items of *X *plus those of *Y *must be less than those of *Z*. This can be simply stated as:

3 * *X *+ 4 * *Y *+ 5 * *Z *≤ 40, *X *+ *Y *<*Z*

We have modeled a sort of *knapsack *problem using 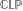
(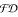). In general, in the modeling stage we can use constraints as well as declarative programs involving them.

Solution's search is performed by a *constraint solver *that is available in the language. The constraint solver uses constraints for sensibly pruning the search tree. One of the main capabilities is called *constraint propagation*. Constraint propagation reduces the domains of the variables eliminating those values that cannot lead to constraint solutions. For instance, in the considered example, constraint propagation reduces the domains of the variables *X*, *Y*, and *Z *to {1, ..., 4}, {1, ..., 4}, and {3, ..., 6}, respectively. For finding a possible solution, a further built-in capability – the labeling predicate – can be used. We can look for a generic solution as well as for a solution minimizing some function. In the example above, we could ask for minimizing the function -2*X*^2 ^+ *Y *+ 4*Z*. This can be done by adding a constraint of the form:

*F *= -2 * X * *X *+ *Y *+ 4 * *Z*, labeling([minimize (*F*)], [*X*, *Y*, *Z*]).

The constraint solver then exploits the solution's search using constraint propagation and branch-and-bound techniques returning the answer:

*F *= 3, *X *= 3, *Y *= 1, *Z *= 5

The library clpfd of SlCStus Prolog [17] allows to effectively program in this framework. Let us observe that it is not required that *F *be a linear function.

The above described approach to optimization combinatorial problems is the so-called *Constrain & Generate *technique introduced as opposed to the *Generate & Test *technique of the classical Logic Programming approach (see, e.g. [18]). In the latter approach, a first phase generates non-deterministically a possible solution, and then the deterministic test-phase checks whether the solution is acceptable or not. If the search space is exponential, this technique is not applicable. In the former approach, a first deterministic phase introduces a number of constraints, then a non-deterministic phase starts the generation of the solutions' space. The constraints introduced allow to sensibly prune the solutions' space in order to make the procedure effective. Moreover, in this phase one can take advantage from language built-in strategies (such as constraint propagation, branch and bound) and it is possible to further drive the solution search by means of problem-dependent heuristics.

**Figure 1:**
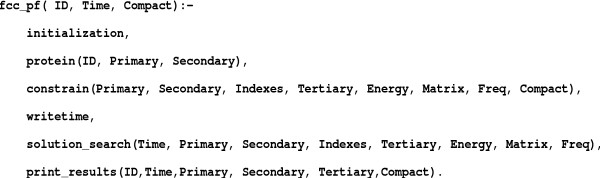
Main program predicate.

We have followed the Constrain & Generate programming style for encoding the protein structure prediction problem. As a matter of fact, the main predicate of our solution is of the form reported in Figure 1.

**Figure 2:**
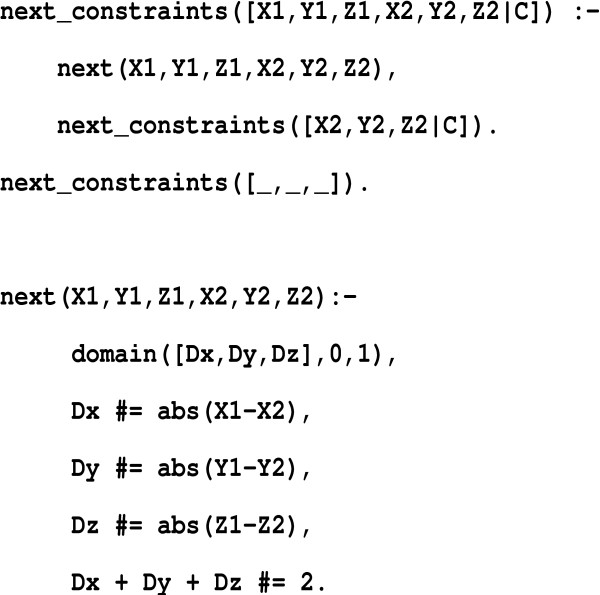
Code for stating that consecutive aminoacids must be in adjacent lattice points.

In the definition of the predicate constrain the protein structure prediction problem is modeled using constraints. In particular, the energy function is encoded in the Energy parameter, The predicate solution_search is aimed at looking for the solution minimizing the Energy parameter. The other predicates are auxiliary predicates. initialization resets some parameters, protein recovers the relevant input (see also **Methods **Section), writetime and print_results are output predicates. The constraint predicate is defined using several predicates each of them modeling one of the properties of the problem. For instance, the predicate next_constraints sets the distance between consecutive aminoacids (see Figure 2).

Briefly, next_constraints recursively calls the predicate next for each pair of consecutive aminoacids. Assume that <*X*1, *Y*1, *Z*1> and <*X*2, *Y*2, *Z*2> are the variables that will store the positions of a consecutive pair of aminoacids, then the predicate next states that |*X*1 - *X*2| + |*Y*1 - *Y*2| + |*Z*1- *Z*2| = 2 and that |*X*1 - *X*2| ∈ {0, 1}, |*Y*1 - *Y*2| ∈ {0, 1}, |*Z*1 - *Z*2| ∈ {0, 1}. This is exactly the notion of adjacency in the face-centered cubic lattice of size 2 that we have used (see also the **Methods **Section).

## Results and discussion

### Constrained optimization problem in 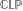
(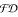)

In Table 1 we report the results of the experiments with the 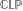
(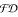) code described in the **Methods **Section. All tests are done using SICStus PROLOG 3.11.1 [17] and a PC P4, 3.06 GHz. The structures of the protein model systems analyzed are known and stored in the PDB [3]. In the protein model systems 1LE3, 1PG1, and 1ZDD terminal protecting groups have been neglected.

From left to right, the meaning of each column is as follows: the protein PDB identification code, the number *N *of aminoacids, the execution time, the energy of the best model found and its RMSD from the native structure for all the residues and for the core residues only. When there is not explicitly written "limit" it means that the program successfully terminated in the time reported; otherwise the program terminated due to time limit. We wish to observe that the results with time limit 10 h/24 h are typically computed in few hours. The rest of the time is used to further explore the solutions' space.

When a CF = *η *is reported a further constraint on the compactness ratio *η *is added before the search. CF = *η *bounds the linear distances |*X*_*i *_- *X*_*j*_|, |*Y*_*i *_- *Y*_*j*_|, and |*Z*_*i *_- *Z*_*j*_| between all pair of residues *i *and *j *to *ηN *where *N *is the length of the primary list. If *η *is low (e.g. 0.2), this constraint imposes a compact form to the protein and strongly reduces the running time.

One of the structural constraints considered is the presence of disulfide bonded residues (ssbonds). The rigid structure of the lattice is such that a low value of Euclidean distance (e.g., 2) between ssbonds often precludes all possible solutions. For this reason the default is chosen as 6. However, in some cases we tried computations with lower value. In these cases in the table the text *ss *= *γ *is reported.

The secondary structure, as computed from the deposited structure in PDB, has been input as constraint. As a unique exception, in the case of 1VII(*) we have instead predicted it using the *GOR IV *secondary structure prediction method [19].

**Figure 3:**
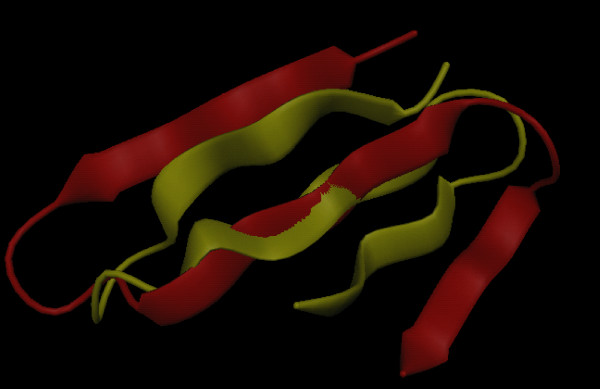
Native (yellow) and 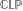 model after all-atom reconstruction and optimization (red) for WW domain (PDB id. 1E0M). The trace of core residues (7–30) is shown.

The predicted structures have been also transformed into all atoms models as described in the **Detailed models from lattice models **Section. There is some improvement in general on RMSD from native structure. This is especially significant when the starting structure is already close to the native one, being not merely due to increasing compactness of the structure. It is moreover reassuring that the procedure we are discussing is able to recover realistic models starting from the very simplified lattice models. The RMSDs of the resulting detailed models from the corresponding native structures are reported in Table 2. In order to assess the quality of the detailed model, the trace of the native structure and the reconstructed and optimized all-atom model are shown in Figure 3 for the core residues (7 to 30) of the WW domain (PDB id.: 1E0M).

We conclude the section comparing some results of our prediction with those returned by the well-known HMMSTR/Rosetta Prediction System [20]. This program does not use a lattice as underlying model: aminoacids are free to take any position in ℝ^3^. For the sake of comparison, we have used it as an *ab-initio *predictor (precisely, we have disabled the *homology *and *psi-blast *options). The comparison is obviously not fair because in our case secondary structure is known and not predicted. Times are obtained from the result files, but it is not clear to which machine/CPU occupation they refer. Results are reported in Table 3. HMMSTR/Rosetta prediction runs presumably faster, but our predictions (which however include known secondary structure) improve the RMSD (except for one case).

### Constrained molecular dynamics simulation

We have used secondary structure information in conjunction with the well-established methodology of molecular dynamics simulations in order to implement a procedure similar to the one implemented using 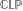 on the 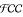 lattice. Secondary structure elements have been imposed through a constraining potential as described in the **Methods **Section. In order to search the conformational space a simulated annealing procedure has been adopted. Globularity of the simulated proteins is forced by a harmonic constraint on the radius of gyration.

The simulation time, ranging approximately between one and four CPU days, required for folding each protein on a 1.533 GHz AMD Athlon processor is reported in Table 2. The columns (from left to right) in Table 2 report the PDB identification code of the protein, the number of residues, the RMSD from native structure computed on C_*α *_atoms on the whole protein and only on core residues and the simulation time. The last column reports the RMSD from native structure for models obtained by 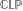 after addition of all atoms and energy minimization as described in the **Methods **Section.

The simulation time needed for obtaining structures similar to native structures increases with the size of the protein both for the increasing size of the system and for the longer simulated annealing runs needed because of increasing complexity of the free energy landscape. Unfortunately a safer scheme would employ substantially longer simulation times.

This fact prompts for searching alternative ways to employ the same ideas.

The results in terms of RMSD from native structure support the idea that folding may be achieved, at least in simulation, by a hierarchical approach where local secondary structure elements are formed first and later their arrangement and contacts are optimized. A similar conclusion has been reached using a different model by Maritan and coworkers [21]. The RMSD on core residues is, in all but one case, less than 5.0 Å. In four out of six cases the RMSD on core residues is close to 4.0 Å. In the worst case, which is also the longest simulated chain, the RMSD on core residues is 7.1 Å.

## Conclusions

The purpose of the present work was to demonstrate that the protein folding problem can be approached by a well-established programming paradigm like 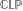. With respect to the few applications reported in the literature so far using the same methodology [22], mainly on the HP protein model [23,24], the present work takes a step further towards more realistic modeling. Notwithstanding the use of a protein simplified lattice model with a simple contact potential realistic models for a few small proteins have been generated by using 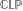. In the present application the known secondary structure of the protein has been imposed as a constraint. 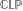 has been applied on face centered cubic lattice models of proteins where every aminoacid is represented by a single point on the lattice that can take one out of six possible positions with respect to the previous three aminoacids. It is immediately seen that the time needed for a systematic space search for such model grows exponentially with the number of free aminoacids. 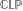 is a programming paradigm that is suited for the solution of optimization combinatorial problems. In 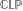 the problem and the related heuristics are extremely natural to be programmed. Moreover, the constraint propagation allows to control the search in the huge solution's space.

The results obtained using this approach and reported in Tables 1 to 3 show that for small proteins a solution for the optimization problem is obtained in less than few hours. For the larger proteins studied here the inaccuracies of both the lattice model and contact potential prevent finding a compact solution. These problems are more likely to appear with increasing size of the protein and when the length of non-constrained chain connecting two secondary structure elements is short, because the lattice allows a limited set of conformations.

Further work is being devoted towards a more realistic modeling representation of the protein, with at least two centers of interaction per residue, and towards refinement of the potential function by including a term for rotamer preferences. This term should map on the lattice the directional preferences of each unit with respect to the previous three units. Each of the six possible next positions for each unit should be weighted by an energy term derived from database analysis.

Also the optimal size of non constrained parts of the chain will be determined in order to allow more possible relative orientations among constrained secondary structure elements, possibly without increasing significantly the computation time. At present, however, when the positions of all atoms are reconstructed from the lattice C_*α *_trace, the RMSD on core residues of the resulting models, after energy minimization, compared to native structures, is as low as 4.8 Å for the thermostable domain of villin headpiece (PDB id.: 1VII), 3.6 Å for the WW domain (PDB id.: 1E0M), 2.3 Å for the coat protein-binding domain of bacteriophage P22 (PDB id.: 2GP8).

It should be also noted that both the thermostable domain of villin headpiece and the WW contain three secondary structure elements that can be arranged in different ways in order to produce a compact structure. The low RMSD is therefore significant.

A comparable protocol employing a molecular dynamics simulated annealing procedure still leads to superior results for larger proteins, as expected because the protein representation is more accurate, but it takes longer execution times between one and four days on a 1.5 GHz P3 machine.

Recent results have shown that simplified models and more refined models can be employed successfully in hierarchical modeling procedures [9,10]. The results obtained in the present work suggest that 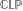 could be useful for finding starting conformations for further refinement.

## Methods

### The protein structure prediction problem as a minimization problem

The sequence of aminoacids defining a protein is called *primary structure*. This structure uniquely determines the (3D) native conformation, also known as *tertiary structure*. The *protein structure prediction problem *is the problem of predicting the tertiary structure of a protein given its primary structure. The native tertiary structure minimizes the global free energy of the protein.

### Abstraction level

We consider each aminoacid as a single *sphere *centered in its *C*_*α *_atom; the distance between two consecutive *C*_*α *_atoms is assumed to be 3.8 Å Recent results (see, e.g., [13]) show that a contact between two residues, when represented only by their *C*_*α *_atoms, is optimally defined for *C*_*α *_- *C*_*α *_distances shorter than 6.4 Å The number is obtained as the sum of the radius of the two *C*_*α *_carbon atoms we are dealing with (2 x 1.9 Å) and the value of 2.6 Å empirically determined in [13] for van der Waals surface contact. A table that points out the energy associated to pairs of aminoacids in *contact *has been developed [12,13]. Let us denote by Pot(*x*, *y*) the energy value associated to a contact between aminoacids *x *and *y *(the order is immaterial); this value can either be positive or negative, according to the pair *x*, *y*.

### Lattice model

According to [25] we use the *Face-Centered Cubic Lattice *(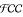) that allows realistic angles between consecutive residues. The lattice is composed by cubes of size 2, where the central point of each face and the vertices are admitted. Thus, the domain 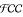 consists in a set of triples <*x*, *y*, *z*> where <*x*, *y*, *z *∈ >. We recall that given a point <*x*, *y*, *z*>, its *2-norm *is: ||<*x*, *y*, *z*>|| = 
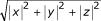
. Given two points *p*_1 _and *p*_2_, ||*p*_1 _- *p*_2_|| is known as their *Euclidean distance*.

Going back to the 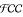 lattice, two points at Euclidean distance 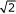 are linked together, forming a *lattice unit*, corresponding to the distance of 3.8 Å. In this lattice, each point is adjacent to 12 neighboring points. A *contact *is defined between two non adjacent residues placed on two vertices of a side of a cube (i.e. they have Euclidean distance equal to 2, corresponding to 5.4 Å). This number can be considered a good approximation of the limit of 6.4 Å described above.

### Mathematical formalization

In this setting, it is possible to formalize the protein folding problem as an optimization problem. Given a sequence *S *= *s*_1 _... *s*_*n*_, with *s*_*i *_aminoacids, a *fold *of *S *is a function *ω *: {1, ..., *n*} → 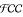 such that: ||*ω*(*i*) - *ω*(*i *+ 1)|| = 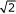 and ||*ω*(*i*) - *ω*(*j*)|| ≥ 2 for *i *≠ *j*. The first constraint states that consecutive aminoacids have a fixed distance, corresponding to one lattice unit; the second that each aminoacid occupies a unitary sphere and that two spheres cannot overlap.

The protein folding problem can be reduced to the optimization problem of finding the fold *ω *of *S *such that the following energy is minimized [26,27]:


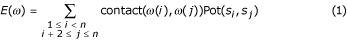


where contact(*ω*(*i*), *ω*(*j*)) is 1 if ||*ω*(*i*) - *ω*(*j*)|| = 2, 0 otherwise. To avoid solutions equivalent modulo simple symmetries, other constraints can be added on the first positions.

### Complexity issues

The decision version of this problem (and even of its HP-abstraction) is proven to be NP-complete on various lattices [28,29]. However, we do not want to solve the problem for proteins of arbitrary length. Solving it for length *N *= 200–300 could be considered as an important contribution to biological sciences and there are yet such results using the HP-abstraction [30]. Thus, in spite of its NP-completeness, it is important to understand the size of the solution's space. The size of the solution's space is the number of *self-avoiding walks *on the 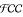 lattice that can be approximated by the formula (cf., e.g., [31])

*SAW*_*fcc *_= 1.26*N*^0.162^(10.0364)^*N *^    (2)

This formula should modify in the presence of additional constraints as mentioned later.

### Main implementation issues

**Figure 4:**
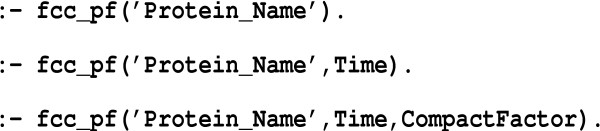
Program execution modes.

Our implementation of the protein folding minimization problem described in the above sections is based on the code briefly introduced in the **Background **Section. The complete program and related material can be found in [32]. The program consists of ~2000 lines and, once loaded in SICStus Prolog, one may call goals of the kind reported in Figure 4, where Protein_Name is a standard PDB identification code, such as 1ENH. Time is the maximum amount of time in seconds that we let to the runtime; the default is 10 hours. CompactFactor allows to impose an additional constraint on the maximal distance between every pair of aminoacids. The rationale behind this additional constraint stems from the observation that protein structures are more compact than expected based on a freely rotating chain model [33]. In particular, the average end-to-end distance for a freely rotating chain model is approximated by 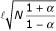 where ℓ is the length of each unit and *α *is the cosine of the angle made by each unit with the direction of the preceding unit. The average end-to-end distance is clearly related to the average maximal dimension of the chain. Based on a survey of protein structures Huang and Powers derived the following approximated formula for the radius of gyration (in Å): 2.2*N*^0.38 ^[34]. Note that the exponent is less than 0.5 which is an underestimate of the exponent for a self avoiding walk. For a uniform density sphere the diameter is 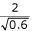 the radius of gyration. The default value for CompactFactor was therefore assumed to be approximately equal to 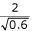 times the radius of gyration which in turn was computed by the empirical formula 2.2*N*^0.38 ^[34].

**Figure 5:**
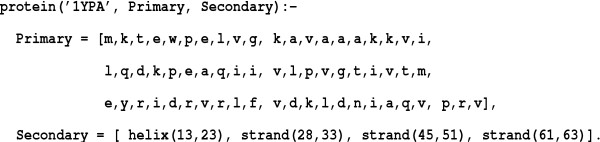
Protein sequence and secondary structure representation

The auxiliary file data.pl stores the Primary and Secondary structures of the proteins that one wishes to test, as, for instance in the example reported in Figure 5. The output in standard PDB format [3] is printed either on the screen or in a file named output-Protein_name.pdb.

### Constraints

The intrinsic complexity of the problem forces us to introduce several other constraints. For instance, we constrain the sum of the coordinates of each aminoacid in the 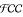 lattice to be even (a property of the 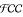 lattice) and we add some constraints for avoiding equivalent symmetric solutions. In what follows, we refer to predicate names as used in the code. avoid_symmetries removes redundant admissible conformations equivalent to others modulo some symmetries and/or rotations. The predicate assigns immediately three consecutive aminoacids positions (in the Tertiary list).

With distance_constraints, we also impose that two non consecutive residues must be separated by more than one lattice unit, to reflect the steric interaction between the *C*_*α*_s modelling aminoacids.

As described above, compact_constraints imposes that, for every pair of aminoacids, the norm of the projection of their distance on each *x*, *y*, *z *coordinate, is smaller than CompactFactor × N.

Further constraints are related to angles. In the 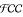 lattice, the angle between three consecutive residues can assume values in {60°, 90°, 120°, 180°}. In real proteins, steric occupancy and energetic potential show a clear distribution of bend angles in the range 90°–150° [7,35]. When transferring on 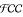 lattice, it is a good approximation to exclude 60° and 180° angles, as unfeasible. This constraint allows us to restrict the search space from a number close to 10^*N *^(cf. formula (2)) to a number close to 5^*N*^.

As said in the **Lattice model **Section, a *contact *is generated by two non consecutive aminoacids with Euclidean distance less than or equal to 2. As a consequence of the constraints applied, it suffices to check for a contact when the lattice distance equals 2, since distance_constraints excludes from the domain the possibility to place two non consecutive aminoacids at one lattice unit.

We also impose constraints coming from secondary structure information. Secondary structure can be predicted with good approximation (e.g., [36]). In our set of data we have collected such information from the Protein Data Bank. We represent secondary structure information as helix(*i*, *j*): elements *i*, *i *+ 1, ..., *j *of the input sequence form an *α*-helix; strand(*i*, *j*): elements *i*, *i *+ 1, ..., *j *are in a *β*-strand; ssbond(*i*, *j*): there is a disulfide bridge between element number *i *and *j*.

We use an auxiliary list called *Indexes *that stores torsional angles defined by four consecutive aminoacid positions. Due to 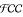 lattice structure and our constraints, every four consecutive aminoacids can form only 6 discrete angles. Thus, each variable in Indexes can assume a value *i *from {0, ..., 5}, representing torsional angles of 0°, 60°, 120°, 180°, 240°, 300°, respectively. With these conventions, helices are approximated by sequences of indexes of the form 5, 5, 5, ... while *β*-strands are associated to sequences of the form 3, 3, 3, .... Note that specifying the coordinates of three points (i.e. to place and orient the protein) and the indexes, uniquely determines the conformation, ssbond(*i*, *j*), introduces a maximum distance constraint between the aminoacids *i *and *j*. The predicate energy_constrain is developed using an auxiliary symmetric matrix *M*. The optimal fold is reached when the sum of *M *elements is minimal. During the *labeling *phase, the information stored in *M *is used to control the minimization process and to cut the search tree.

### Labeling stage

To reduce the size of the solution's space visited during execution, we have replaced the built-in labeling predicate with an ad-hoc constraint-based solution search predicate, called **solutions_search**. We describe here briefly the main features of this predicate and of its auxiliary predicates.

**solutions_search** • If the **Tertiary list** or the **Indexes** list is ground (already computed), then it terminates the folding process (possibly, after a call to the built-in labeling).

• Otherwise, it calls **choose_labeling**. When this procedure terminates, it calls recursively **solutions_search**. Termination is guaranteed by the fact that each call to **choose_labeling** reduces the number of non-ground variables.

**choose_labeling** • If the number of variables to be instantiated is low (in our code less than 4), it calls the built-in labeling.

• Otherwise, it calls **selection_strategy**. This predicate computes several subsequences of the list of **Indexes**. Each subsequence consists of alternations of ground elements and non-ground variables. **selection_strategy **selects the *most known subsequence*, namely the one containing the smallest ratio of variable over ground indexes, preferring the ones that include a ssbond. If in the selected subsequence there are too many variables, an arbitrary subsequence cut is done. After the subsequence is selected, the procedure **labeling_new_launch** is called.

**labeling_new_launch** It calls the auxiliary predicate** labeling_new** but stops the solution search when the global runtime is greater than the input time limit. If this is the case, the best computed solution is returned.

**labeling_new** This procedure receives the chosen sublist to be folded. Each index variable in it, is assigned an admissible value between 0 and 5. The order of values that is tried for each index is described by a pre-computed auxiliary list. For each torsional index, a frequency statistics of the 6 indexes is pre-computed and extracted from the PDB, according to the specific aminoacid sequence involved locally. We use this information to direct the search and explore first the most common torsional angles, in the hope that this selection rule reflects nature's strategy.

Moreover, the energy associated to the fold is minimized. For doing that, after each instantiation of a fixed number *t *of variables in a phase, we collect the best known ground admissible solution, its energy and its associated potential matrix. We compare the current status to history and decide if it is reasonable to cut the search tree. In particular, we designed a heuristic that allows to control the effectiveness of the cut, adapting it dynamically to the status of the fold. Practically, when the protein is partially specified, we estimate the ratio between ground and non-ground variables in the potential matrix. If the ratio is low (i.e. the protein is poorly determined), we allow the current energy to be worse than the corresponding counterpart in the best fold so far reached. When the ratio is high (i.e. protein almost folded) we constrain the current energy to be slightly lower than the previous best known.

### Molecular dynamics simulations

In order to have a fair comparison with a similar approach using all-atom protein models we built detailed all atom models for six proteins in the studied set (namely those with PDB id. code: 1VII, 1E0M, 2GP8, 1ENH, 2IGD, 1YPA) and imposed, through torsional constraints, the secondary structure geometry found in the native structure. The constraining potential was 100 * (*θ *- *θ*_0_)^2 ^kcal/(mol rad^2^). The reference target angles (i.e. *θ*_0 _in the previous formula) were set to *φ *= -139 and *ψ *= 135 for residues in *β*-strand and to *φ *= -48 and *ψ *= -57 for residues in *α*-helices. For all constrained residues also the *ω *dihedral angle was constrained at 180 degrees.

The chain was first built fully extended and minimized by 400 steepest descent minimization steps and by 500 conjugate gradients minimization steps.

The protein was then heated in 10 ps up to 900 K in 20000 steps using a timestep of 0.0005 ps. Then the temperature was lowered down to 270 K in 20 steps. During each step molecular dynamics simulation was carried out for 100 ps for a total simulation time of 2 ns.

Simulations used the Generalized Born implicit solvent method [37] as implemented in the program CHARMM [38] with standard parameters for proteins. The forcefield used was CHARMM v.27 [39].

In order to obtain globular protein during simulation a constraint on the radius of gyration (computed only on C_*α *_atoms) was imposed. The target radius was decreased during the simulation from a value proper of an extended conformation down to the value given by 2.2*N*^0.38 ^[34] where *N *is the number of residues.

The potential used for enforcing compactness was: 
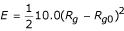
 kcal/mol, where 
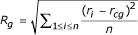
, *n *is the number of atoms, *r*_*cg *_is the center of geometry of the same group of atoms, and *R*_*g*0 _is the target gyration radius which is decreased during simulated annealing down to the theoretical value based on the formula cited above.

### Detailed models from lattice models

The models obtained by 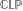 described here may be converted into all-atom models which are realistic models of proteins. As a test the structures of all the proteins tested by simulated annealing described above were converted using the Maxsprout server [40] into an all heavy atom model. Hydrogens have been added using the module HBUILD in the program CHARMM [38] and the resulting structure was relaxed by energy minimization (using a distance dependent dielectric constant). First a minimization was performed with all backbone atoms fixed, then only C_*α *_atoms were fixed and finally a 100 ps molecular dynamics simulation (following a heating phase of 10 ps) using the Generalized Born implicit solvent model was performed. The resulting structure at the end of the simulation was energy minimized.

The initial minimizations required 1500 minimization steps each, because the starting structures were built from the lattice models. The final minimization, on the structure relaxed by molecular dynamics simulation, employed 900 minimization steps. During molecular dynamics simulation the radius of gyration and backbone torsion angles corresponding to residues constrained in the 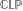
(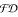) procedure were constrained as described above.

## Figures and Tables

**Table 1:** Experimental results

Name	N	Time	Energy	RMSD
1LE0	12	1.3 s	-9040	2.8 / 2.6 (2–11)
1KVG	12	7.3 s	-14409	2.7 / 2.4 (3–11)
1LE3	16	2.3 s	-13653	3.0 / 2.7 (2–1 5)
1EDP	17	20.4 s	-19389	4.3 / 1.1 (9–15)
1PG1	18	14.6 s	-10126	6.0 / 5.2 (4–17)
1ZDD	34	300 s (limit)	-20412	5.6 / 5.6 (5–34)
		17 m25 s	-22350	4.0 / 4.0 (5–34)
1VII	36	300 s (limit)	-20860	10.4 / 6.7 (4–32)
		1000 s (limit)	-22377	9.1 /6.3 (4–32)
		7 h42 m	-26408	10.2 / 7.8 (4–32)
	CF = 0.3	3 h58 m	-28710	8.0 / 7.4 (4–32)
1VII(*)	36	300 s (limit)	-17948	9.2 / 7.3 (4–32)
		1000 s (limit)	-17948	9.2 /7.3 (4–32)
		3 h20 m	-21211	10.3 / 6.9 (4–32)
1E0M	37	300 s (limit)	-13830	6.5 / 5.8 (8–29)
		1200 s (limit)	-24613	8.4 / 3.6 (8–29)
		10 h (limit)	-26592	8.8 / 3.4(8–29)
		24 h (limit)	-30163	8.9 / 4.4 (8–29)
2GP8	40	300 s (limit)	-10303	10.5 / 8.9 (6–38)
		1000 s (limit)	-24748	4.1 / 3.5 (6–38)
		10 h (limit)	-26196	4.9 / 3.5 (6–38)
		10 h39 m	-26196	4.9 / 3.5 (6–38)
1ED0	46	300 s (limit)	-29970	7.3 / 4.1 (3–40)
		1000 s (limit)	-32369	8.6 / 7.1 (3–40)
		9 h38 m	-38218	8.0 / 7.2 (3–40)
1ENH	54	300 s (limit)	-12480	10.4 / 8.9 (8–52)
		1000 s (limit)	-12480	10.4 / 8.9 (8–52)
		10 h(limit)	-23373	9.9 / 8.6 (8–52)
		24 h (limit)	-23373	9.9 / 8.6 (8–52)
6PTI	58	300 s (limit)	no sol.	
		1000 s (limit)	-29709	10.0 / 9.7 (3–55)
		10 h (limit)	-37837	10.0 / 9.7 (3–55)
		24 h (limit)	-37837	10.0 / 9.7 (3–55)
	CF = 0.25	48 h (limit)	-42096	9.7 / 9.4 (3–55)
	CF= 0.18	24 h (limit)	-47451	10.9 / 10.7 (3–55)
2IGD	60	300 s (limit)	-24158	19.3 / 16.3 (6–59)
		1000 s (limit)	-29178	19.0 /16.2 (6–59)
		10 h (limit)	-37479	16.9 / 15.0(6–59)
		24 h (limit)	-37479	16.9 / 15.0 (6–59)
	CF = 0.17	4 h 59 m	-40588	12.6 / 11.5 (6–59)
2ERA	61	300 s (limit)	-28993	11.6 / 10.6 (3–55)
		9 m28 s,	-30746	12. 3/ 12.1 (3–55)
	ss = 5	15 m13 s	-31692	12.7/11.6 (3–55)
	CF = 0.25, ss = 5	15 m12 s	-33693	10.9/9.3 (3–55)
	CF = 0.25, ss = 4	1000 s (limit)	-32985	12.3/12.4 (3–55)
	CF = 0.19, ss = 5	1000 s (limit)	-34275	10.6/8.9 (3–55)
	CF = 0.19, ss = 4	1000 s (limit)	-38138	11.6/10.6 (3–55)
1SN1	63	300 s (limit)	no sol.	
		1000 s (limit)	-53888	13.0 / 10.5 (2–51)
		10 h (limit)	-57615	11.9/ 9.6 (2–51)
	CF = 0.25, ss = 5	10 h (limit)	-47121	8.6 / 8.1 (2–51)
1YPA	63	300 s (limit)	-36626	16.1 / 9.4 (12–52)
		1000 s (limit)	-33886	17.1 / 10.9 (12–52)
		10 h (limit)	-33886	17.1 / 10.9 (12–52)
	CF = 0.17	100 s (limit)	-26297	12.5 / 10.5 (12–52)
	CF = 0.17	10 h (limit)	-60244	12.9 / 9.8 (12–52)

**Table 2:** Summary of molecular dynamics results

Name	N	RMSD (Å)	Time	RMSD ( 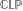 + opt) (Å)
1VII	36	5.3 / 4.7 (4–32)	17.8 h (2 ns)	5.8 / 4.8 (4–32)
1E0M	37	5.5 / 4.0 (7–30)	26.3 h (4 ns)	8.7 / 3.6 (7–30)
2GP8	40	5.9 / 3.8 (6–38)	37.7 h (4 ns)	3.9 / 2.3 (6–38)
1ENH	54	5.9 / 5.0 (8–52) / 3.7 (8–36)	29.4 h (2 ns)	11.2 / 10.7 (8–52) / 4.7 (8–36)
2IGD	61	5.7 / 4.1 (6–59)	48.6 h (4 ns)	12.9 / 11.5 (6–59)
1YPA	64	9.2 / 7.1 (12–52)	116.9 h (8 ns)	11.8 / 9.4 (12–52)

**Table 3:** Comparison with Rosetta predictions

Name	N	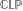 Time	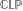 RMSD	Rosetta Time	Rosetta RMSD
1ZDD	34	17 m.25 s.	4.0	5 m.35 s.	3.5
1VII	36	3 h.58 m.	7.4 (4–32)	5 m.35 s.	4.2
1E0M	37	10 h.	3.4 (8–29)	6 m.35 s.	7.7
2GP8	40	10 h.	3.5 (6–38)	6 m.35 s.	6.4
1ED0	46	10 h.	7.2 (3–40)	7 m.23 s.	8.9
